# Very Early Corona Treatment-Mediated Artificial Incubation of Silkworm Eggs and Germline Transformation of Diapause Silkworm Strains

**DOI:** 10.3389/fbioe.2022.843543

**Published:** 2022-02-11

**Authors:** Yu-Li Zhang, Yang Huang, Ping-Yang Wang, Qiang Li, Li-Hui Bi, Ai-Chun Zhao, Zhong-Huai Xiang, Ding-Pei Long

**Affiliations:** ^1^ Sericultural Research Institute, Guangxi Zhuang Autonomous Region, Nanning, China; ^2^ State Key Laboratory of Silkworm Genome Biology, Key Laboratory for Sericulture Functional Genomics and Biotechnology of Agricultural Ministry, Southwest University, Chongqing, China; ^3^ Department of Biology, Georgia State University, Atlanta, GA, United States; ^4^ Department of Mathematics and Statistics, Georgia State University, Atlanta, GA, United States

**Keywords:** *Bombyx mori*, corona treatment, egg diapause, germline transformation, insect, artificial incubation

## Abstract

Diapause is an important biological characteristic for many insect species to adapt to adverse environmental conditions and maintain the continuity of the race. Compared with the traditional hydrochloric acid or/and cold storage treatment methods, the artificial corona incubation technology of silkworm (*Bombyx mori*) eggs has many advantages including, the absence of pollution, easy operation and safety. However, this technology has not yet been applied in sericulture. In this study, we developed a novel artificial corona instrument to successfully disrupt the diapause of newly laid and refrigerated eggs from various Chinese and Japanese lineage silkworm strains. Subsequently, we invented a very early corona treatment (VECT) strategy to prevent the diapause of newly laid silkworm eggs within 4 h of oviposition. The hatching rates of the larvae were more than 95% in all diapause silkworm strains, which was comparable to the effect of the traditional HCl treatment strategy. In addition, we developed a combination strategy of VECT and pre-blastoderm microinjection and successfully created transgenic silkworms in various diapause strains. The results of the current study can aid in improving the corona artificial incubation technology and promote its application in sericulture.

## Introduction

Diapause, a reversible developmental arrest, is an important physiological characteristic of insects to cope with periodic adverse environmental conditions and maintain ethnic continuity. Environmental conditions such as photoperiod (Emerson et al., 2009), temperature (Nibouche, 1998) and food quality (Wallace, 1970) trigger physiological responses that induce diapause in insects, and the internal conditions are mainly regulated by hormones (Nijhout, 1998; Sim and Denlinger, 2013). Diapause can be categorised based on the life stages of insects suspending their development. Different insect species have varying diapause stages, including early embryo or egg (Feng et al., 2012), larval (Feng et al., 2012), pupal (Resh and Cardé, 2009), and adult diapause (Xu et al., 2004). *Bombyx mori* is one of the most economically important insects, which exhibits a well-defined embryonic diapause that typically occurs in the late gastrula stage of embryogenesis when the cell cycle is arrested in the G2 phase (Nakagaki et al., 1991). The dormancy of *B. mori* enables it to overcome severe environmental changes such as insufficient food supply and low temperatures (Yamashita and Hasegawa, 1985). It also benefits the copulation of silkworms and ensures the reproduction of the offspring by synchronising populations (Yamashita and Hasegawa, 1985). Diapause is important in sericulture for protection of the eggs and larval hatching. Research on the mechanism of diapause and the release method of silkworms is of significance in the practice of silkworm seed production.

Diapause is determined by genetic factors and can be triggered by hormones in the silkworm (Xiang et al., 2005). Several studies have been conducted to understand the mechanism of diapause and its termination in *B. mori* (Liu et al., 2014; Jiang et al., 2017; Shen et al., 2018). In addition, researchers have considered combination approaches using physical or chemical stimulation to terminate the diapause of silkworm eggs to satisfy the needs of sericulture (Fan et al., 2007; Zhao et al., 2012). Treating silkworm eggs with hydrochloric acid (HCl) or/and cold storage is the most commonly used method to prevent/terminate the diapause of eggs in sericulture (Xiang et al., 2005). A standard method for newly oviposited silkworm eggs is placing them in HCl for several minutes to prevent them from entering diapause and enabling normal hatching. On the other hand, diapause of silkworm eggs can be terminated by soaking them in HCl after a period of cold storage (30–90 days) at 4°C. However, the highly concentrated HCl used in the treatment can pollute the environment and cause health problems in the operators (Xiang et al., 2005). In addition, a few studies have reported that hydrogen peroxide (Shen et al., 2003), dimethyl sulphoxide (Yamamoto et al., 2013) or oxygen (Gong et al., 2016) were used to treat silkworm eggs to prevent the onset of diapause; however, these methods were also associated with disadvantages, such as toxicity, instability, contamination of chemicals and cumbersome procedure. With the development of the economy and society, the above problems became more prominent, which restricted the popularisation and application of these methods in sericulture.

Artificial incubation of silkworm eggs with electric induction was introduced in 1914, followed by the invention of the electric incubation device in 1924, which increased the hatch rate up to 90%. However, the technique did not gain popularity due to shortcomings of the device. Until 1993, Xian and Kobayashi performed the improved corona discharge method to treat the eggs at 20 h after oviposition and obtained a high larval hatching rate (Xian and Kobayashi, 1993). Since then, Chinese researchers have modified and optimised the usage of the corona discharge device, including its technical parameters (e.g. voltage and length of the pole needle) and stimulation conditions (Yang et al., 1994; Ye et al., 1996). It has been demonstrated that corona treatment on newly laid or refrigerated diapausing eggs resulted in similar hatching rates as those following HCl treatment (Chen et al., 1995a; Chen et al., 1995b; Yang et al., 1995; Wu et al., 1997). In addition, there was no substantial difference in the growth and development, survival rate and yield and quality of silkworm cocoons between the two treatments (Ye et al., 1996). Compared with other artificial incubation methods, the corona artificial incubation technique invented in the 1990s has the remarkable advantages of cost-effectiveness, operability and reduced pollution (as caused by HCl treatment). However, due to the limitation of the technology (e.g., uneven discharge of the electrode plate and insufficient power of the corona device) and factors affecting the effectiveness of hatching (e.g., silkworm species and age of the eggs), the corona artificial incubation method faced challenges in its promotion and application (Xu and Yang, 1998).

In this study, we designed a novel artificial corona instrument and used it to successfully disrupt the diapause of newly laid and refrigerated eggs from various silkworm strains (including Chinese and Japanese lineages). The hatching rates of the silkworm larvae were comparable to those following the traditional HCl treatment. Subsequently, we invented a more eco-friendly, safer, less expensive and handier strategy with broad adaptability, named very early corona treatment (VECT), to prevent diapause of silkworm eggs. This strategy could prevent eggs from entering diapause by incorporating corona treatment on newly laid silkworm eggs within 4 h of oviposition. We have systematically studied the effects of the time point, duration and other factors on the hatching rate of silkworm larvae following corona treatment. In addition, this study attempted to combine the VECT with the pre-blastoderm microinjection technology to create transgenic individuals in various diapause silkworm strains for the first time. The VECT strategy invented in this research can theoretically prevent the diapause of all silkworm strains effectively, aid in improving the corona artificial incubation technology and promote its application in sericulture.

## Materials and Methods

### Experimental Animals

Five bivoltine *B. mori* strains were used in this study: 1) Dazao (designated “DZ”, Chinese lineage, native inbred strain); 2) Dong/Ting (designated “DT”, Chinese lineage, commercial parental strain); 3) Bi/Bo (designated “BB”, Japanese lineage, commercial parental strain); 4) 932 (Chinese lineage, commercial parental strain) and 5) 7532 (Japanese lineage, commercial parental strain). DT/BB and 932/7532 were the two pairs of commercial parental strains sourced from silkworm seed propagation farms in Nanchong city (Sichuan, China) and Nanning city (Guangxi, China), respectively. The aforementioned *B. mori* strains were maintained at the Gene Resource Bank of Domesticated Silkworms in Southwest University (Chongqing, China). The eggs were maintained at 25°C in a moist chamber (85%–90% relative humidity) until hatching. The larvae were reared at 25°C (75%–80% relative humidity) and fed mulberry leaves.

### Composition of the Novel Artificial Corona Instrument

GZ-01, the artificial corona instrument developed by us, consists of two parts: a high-voltage direct-current power supply and electrodes for creating corona. The metal case of the high voltage power supply is 20 cm × 12 cm × 12 cm (length × width × height) in size. The adjustable display voltage is 0–15 kV. The power line is connected to a 220 V household alternating current source. Both positive and negative plane electrodes are made of 15 cm × 8 cm stainless steel plates and fixed on the insulating bracket with a base. A single metal pole needle with a length of 1 cm was inserted into the negative plane electrode. The distance between the needle tip and the positive plane electrode (pole pitch) is adjustable.

### Measuring the Range of Corona Produced by the Artificial Corona Instrument

Following mating of the moths from each strain, the eggs were laid on Kraft paper and collected every hour after the start of oviposition. One batch of eggs at 20 h after oviposition was placed on the positive plane electrode and subjected to corona treatment (voltage, 12 kV; pole pitch, 8 mm) for 2 min. These eggs were maintained at 25°C until hatching (approximately 10–14 days), and the diameter range of the hatched silkworm eggs was measured.

### Corona or HCl Treatment Conditions to Prevent the Diapause of Eggs at 20 h After Oviposition

One batch of eggs at 20 h after oviposition (placed on Kraft paper) was divided into three groups: group 1^#^, eggs that were not subjected to any treatment were considered the control; group 2^#^, eggs were placed on the positive plane electrode and were subjected to corona treatment (voltage, 12 kV; pole pitch, 8 mm) for 2 min; group 3^#^, eggs were treated with HCl solution (specific gravity, 1.075) for 5 min at 46°C, following which the eggs were washed in running water and air-dried [An optimized traditional method of HCl treatment (Zhao et al., 2012)]. All eggs were maintained at 25°C until hatching (approximately 10–14 days), and the hatching rate of the larvae was calculated. Each group from one batch contained approximately 100–200 eggs. The eggs were photographed using a stereomicroscope (Olympus MacroViewMVX10-AUTO, Tokyo, Japan) at different developmental stages.

### Corona or HCl Treatment Conditions to Terminate the Diapause of the Refrigerated Eggs

The diapausing eggs of each strain were stored at 4°C for 60 days, following which they were stored at 25°C for 2 h and divided into three groups as follows. Group 1^#^ comprised eggs that were not subjected to any treatment and were considered the control, group 2^#^ comprised eggs that were placed on the positive plane electrode and were subjected to corona treatment (voltage, 12 kV; pole pitch, 8 mm) for 2 min and group 3^#^ comprised eggs that were treated with HCl solution (specific gravity, 1.092) for 5 min 30 s at 47.8°C, following which the eggs were washed and air-dried [An optimized traditional method of HCl treatment (Zhao et al., 2012)]. The steps were the same as those mentioned previously, and the hatching rate of the larvae in each group was finally calculated.

### Optimum Conditions to Prevent the Diapause of Eggs Within 4 h of Oviposition by Very Early Corona Treatment

Following mating of the moths from each strain, one batch of eggs was placed on Kraft paper and collected every 30 min after the start of oviposition. Corona treatment was performed on all eggs based on the same parameters: voltage = 12 kV and pole pitch = 8 mm. The independent variables of the experiment were the developmental stage of the eggs (or the time following oviposition) when treated (eight developmental stages varying from 0.5 h to 4 h following oviposition) and the duration of the corona treatment (eight length of times varying from 5 s to 10 min). Each group from one batch contained approximately 80–150 eggs. The hatching rate of the larvae from each batch was determined following their incubation at 25°C. The hatched larvae were reared into adults, and their physiological indexes, including duration of larval stages, the incidence rate of larvae, cocooning rate, rate of dead cocoons, and larva-pupa rate, were analysed and compared with those hatched by HCl treatment at 20 h after oviposition.

### Germline Transformations of Diapause Silkworm Strains

G0 diapause eggs from each strain were collected for corona treatment under optimised conditions at 2 h after oviposition, following which the eggs were used for microinjection within 6 h of oviposition. Microinjection of the embryos and screening of transgenic silkworms were done as described elsewhere (Long et al., 2012; Long et al., 2013). Briefly, a mixture of *piggyBac*-derived vector pBac (Horn and Wimmer, 2000) and helper plasmid pHA3PIG (Tamura et al., 2000) in ultra-pure water was injected into each egg using a FemtoJet 5247 microinjector system (Eppendorf, Hamburg, Germany). G0 fertile adults were backcrossed with wild-type adults to produce G1 offspring. The G1 individuals were assessed for the expression of green fluorescent proteins under the stereomicroscope with a GFP filter (Olympus MacroViewMVX10-AUTO, Tokyo, Japan), as described previously (Long et al., 2013; Long et al., 2016).

### Statistical Analysis

Data are presented as means ± standard deviation (S.D.) from several separate experiments. Statistical analysis was performed using the Student’s t-test for the comparison of two groups, and the one-way analysis of variance (ANOVA) followed by Dunnett’s multiple comparison test was used for the comparison of more than two groups. A *p* value < 0.05 was considered statistically significant.

## Results

### The Novel Developed Artificial Corona Instrument can Be Successfully Used to Prevent the Diapause of Silkworm Eggs

**FIGURE 1:**
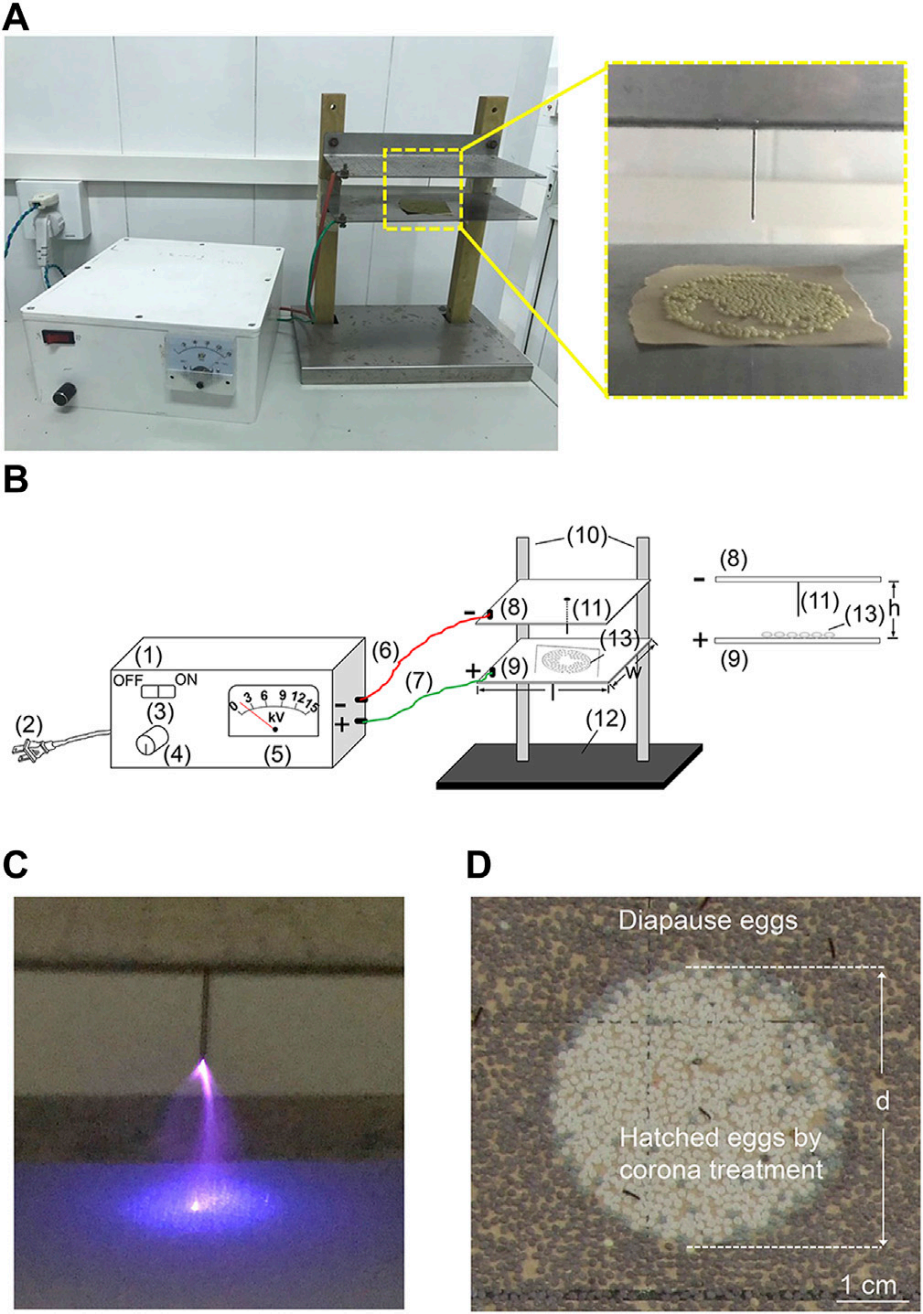
Composition of the self-made GZ-01 artificial corona instrument and the range of action of the corona treatment on silkworm eggs. **(A)** Photographs of eggs processed by the GZ-01 corona instrument. **(B)** Illustration of the GZ-01 corona incubator: 1) metal shell of the high voltage direct current power supply; 2) power plug (connected to a 220 V household alternating current source); 3) power switch; 4) voltage adjusting knob; 5) voltage display (0–15 kV); 6) negative connection line (red); 7) positive connection line (green); 8) negative and 9) positive electrode metal plates (l = 15 cm, w = 8 cm, h = 18 mm); 10) insulation supports; 11) metal pole needle (length = 10 mm, pole pitch = 8 mm); 12) base; 13) Kraft paper covered with eggs. **(C)** Photograph of the corona discharge in weak light. **(D)** Photograph of the effect of corona treatment in preventing the diapause of newly laid DZ eggs. d, the diameter of the circular area of the eggshells.

As shown in Figures 1A,B, the chief feature of our self-made GZ-01 artificial corona instrument is the single metal pole needle at the centre of the negative plate. After instrument debugging and the preliminary experiment, we found that when the output voltage was 12 kV and the pole pitch was 8 mm, an obvious corona discharge phenomenon of the metal pole needle could be observed under weak light (Figure 1C). To test the corona discharge range of the single-pole needle and the effect of different ranges on diapausing eggs, the newly laid eggs of the DZ strain at 20 h after oviposition were subjected to corona treatment for 2 min. The results revealed that the operating range of the corona treatment was a circular area with an average diameter of 41 ± 1.73 mm (*n* = 3), with the hatching rate close to 100%. Surprisingly, almost all the eggs outside the corona treatment range entered diapause (Figure 1D).

### Effects of Corona Treatment in Preventing the Diapause of Newly Laid Eggs at 20 h After Oviposition

**FIGURE 2:**
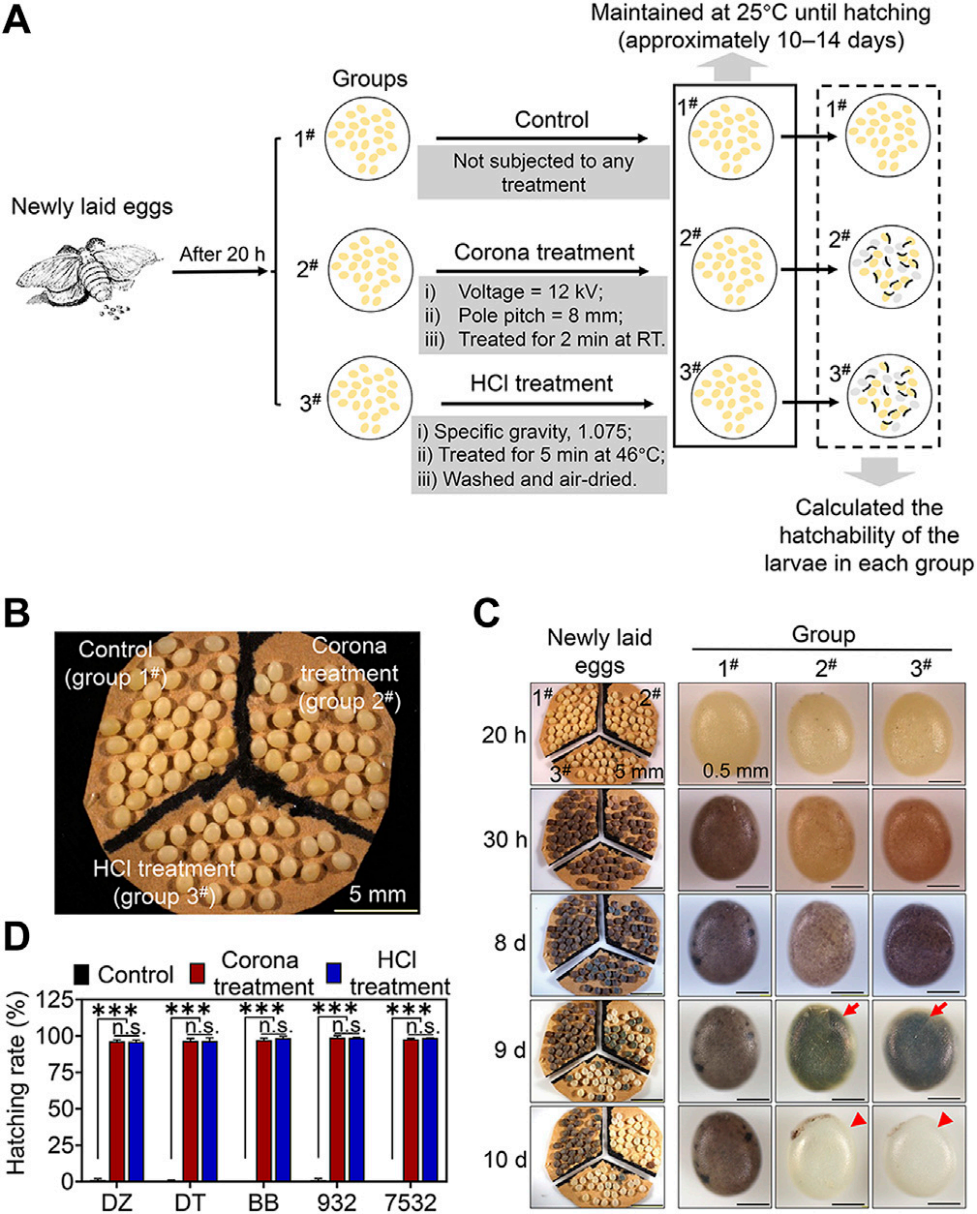
Comparison of the effects of corona and HCl treatments in preventing the diapause of eggs at 20 h after oviposition. **(A)** Schematic diagram of the experimental process of treating newly laid eggs under different conditions and comparing the larval hatchability. **(B)** Image of the grouping of DZ eggs at 20 h after oviposition from one brood. **(C)** Images of the newly laid DZ eggs at different developmental stages from different treatment groups. Red arrows indicate the heads of the larvae developing in the eggs; red triangles indicate the eggshells left by the larvae after hatching. **(D)** The hatching rates of the larvae from different treatment groups of each strain. Error bars represent one standard deviation (*n* = 3; n.s., not significant; ***p < 0.001).

To compare the effects of corona and traditional HCl treatment in preventing the diapause of newly laid eggs at 20 h after oviposition, one batch of eggs (one brood) from each strain were divided into three groups as described in the Materials and methods section and the experimental process is shown in Figure 2A. The eggs of the DZ strain from the control group (1^#^), corona treatment group (2^#^) and HCl treatment group (3^#^) were maintained at 25°C (Figure 2B). The changes in the colour of the DZ eggs were recorded at different development stages, and the final hatching rates of the larvae in each group were calculated. As shown in Figure 2C, at 20 h after oviposition, we did not observe any noticeable difference in the colour of the DZ eggs (light yellow) among the three groups immediately after treatment. In the control group, the DZ eggs turned a red bean colour after 30 h of oviposition and gradually entered diapause. The DZ eggs that had entered diapause completely were greyish purple and did not change colour further. In the corona treatment group, the DZ eggs become a light brown colour after 30 h of oviposition, which gradually deepened until the embryos developed fully and hatched. The change in egg colour in the HCl treatment group was similar to that in the corona treatment group, which gradually deepened with the development of the embryos until the larvae hatched. Furthermore, we observed that the eggs treated using HCl were darker than those subjected to corona treatment at the same developmental stage; however, they were lighter than the diapausing eggs (Figure 2C). This may be due to the different physiological and biochemical reactions of the embryos within the eggs caused by corona or HCl treatment.

The average hatching rates of the larvae from the control group of the five strains were almost zero, irrespective of the Chinese (DZ, DT and 932) or the Japanese (BB and 7532) lineage diapause silkworm strains (Figure 2D and Supplementary Table S1). The average hatching rates of the larvae by corona or HCl treatment from the five strains reached 96.27%–98.9% and 96.05%–98.74%, respectively, and there was no significant difference in the hatching rates between the two treatment groups. These results confirmed that the efficacy of corona treatment in preventing diapause of newly laid eggs at 20 h after oviposition was comparable to that of the traditional HCl treatment.

### Effects of Corona Treatment in Terminating the Diapause of Refrigerated Eggs

**FIGURE 3:**
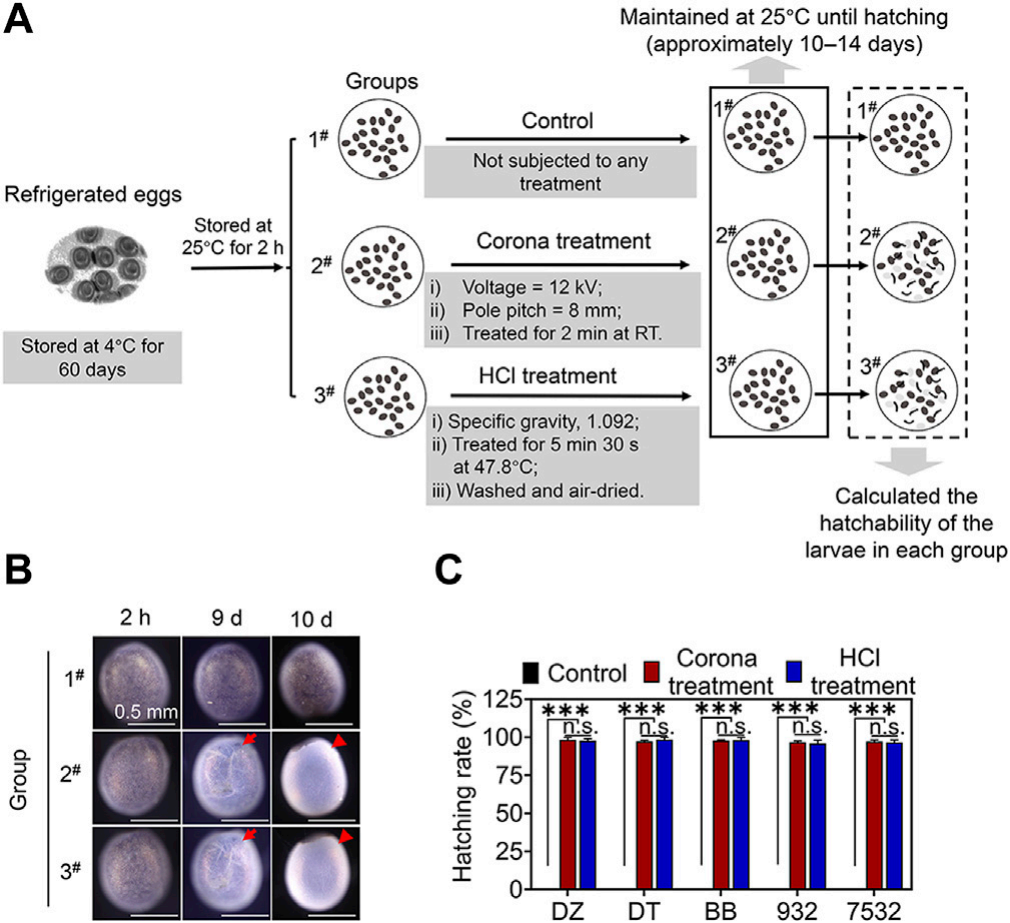
Comparison of the effects of corona and HCl treatments in terminating the diapause of refrigerated eggs. **(A)** Schematic diagram of the experimental process of treating the refrigerated diapausing eggs under different conditions and comparing the larval hatchability. **(B)** Images of the refrigerated diapausing DZ eggs at different developmental stages from different treatment groups. Red arrows indicate the heads of larvae developing in the eggs; red triangles indicate the eggshells left by the larvae after hatching. **(C)** The hatching rates of larvae from the different treatment groups of each strain. Error bars represent one standard deviation (*n* = 3; n.s., not significant; ***p < 0.001).

The experimental process of terminating diapause in refrigerated eggs by corona and traditional HCl treatments is shown in Figure 3A. As described in the Materials and Methods section, the refrigerated diapausing eggs of one brood from each strain were divided into three groups. In the control group, the DZ eggs were maintained at 25°C from 2 h to 10 days and no apparent changes in egg colour were observed, indicating that the diapause of eggs was not broken (Figure 3B). However, in the corona and HCl treatment groups, the eggshells gradually become transparent with the development of embryos, and the larvae hatched and broke out of the shells after 10 days. Statistical analysis revealed that the average hatching rates of the larvae from the control group of the five strains were zero, whereas it reached 96.78%–98.36% and 95.98%–98.48% following corona and HCl treatment, respectively (Figure 3C and Supplementary Table S2). Considering that there was no significant difference in the larval hatching rate between the corona and HCl treatment groups, it may be considered that both treatments have the same effect in terminating the diapause of refrigerated eggs.

### Preventing the Diapause of Newly Laid Eggs Within 4 h of Oviposition by Very Early Corona Treatment

**FIGURE 4:**
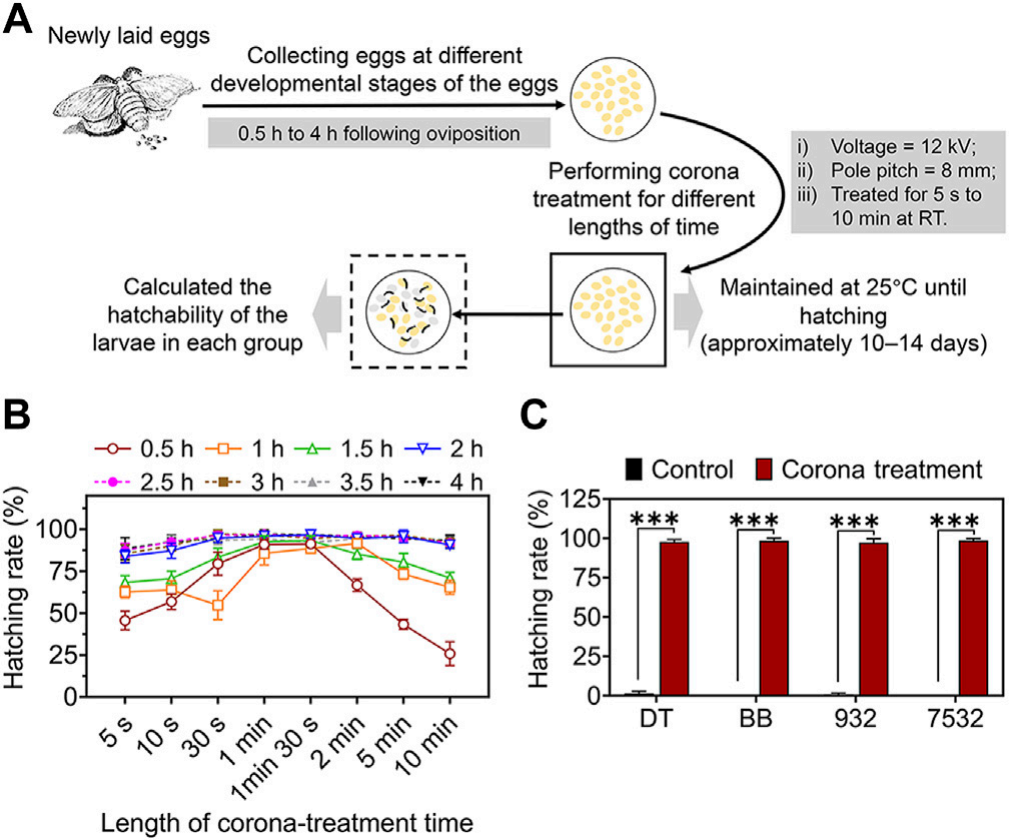
VECT-mediated artificial incubation of eggs within 4 h of oviposition. **(A)** Schematic diagram of the experimental process of treating newly laid eggs within 4 h of oviposition by VECT. **(B)** Line charts of the larval hatching rates of the DZ eggs treated with a combination of different conditions within 4 h of oviposition. Error bars represent one standard deviation (*n* = 3). **(C)** The larval hatching rates of the newly laid eggs from other commercial parental strains following corona treatment for 1 min at 2 h after oviposition. The eggs of each strain that were not subjected to any treatment were considered the control. Error bars represent one standard deviation (*n* = 3; n.s., not significant; ***p < 0.001).

The DZ strain was used to investigate the effect of VECT in preventing the diapause of newly hatched eggs within 4 h by performing corona treatment for different lengths of time at different developmental stages of the eggs. The results of the experimental procedure, shown in Figure 4A, revealed that the larval hatching rates of DZ eggs collected at 0.5–1.5 h after oviposition increased significantly with the variation in the corona treatment times from 5 s to 1 min 30 s. The average maximum hatching rates of the larvae reached 88.57%–93.19% (Figure 4B and Supplementary Table S3). However, when the length of the corona treatment time was longer than 2 min, the larval hatching rates decreased significantly, and the average hatching rates decreased to 25.73%–70.83% following treatment for 10 min. Moreover, the larval hatching rates of DZ eggs collected at 2–4 h after oviposition increased significantly with the variation in corona treatment times from 5 s to 30 s and reached the maximum average value following treatment for 30 s. Subsequently, the larval hatching rates of DZ eggs did not change significantly with an increase in the corona treatment times (from 30 s to 5 min). The maximum average hatching rates of the larvae reached 95.77%–97.15%, which was comparable to the effect of corona or HCl treatment in preventing the diapause of eggs at 20 h after oviposition (Figure 4B and Supplementary Table S3). When the corona treatment time of the DZ eggs collected at 2–4 h after oviposition was increased to 10 min, the larval hatching rates began to decrease. The above results indicated that under the determined conditions of the output voltage (12 kV) and pole pitch (8 mm), the best time to prevent the diapause of eggs within 4 h of oviposition by VECT was 2–4 h, and the most appropriate duration of the corona treatment was between 30 s and 5 min.

Furthermore, eggs from each of the other commercial parental strains were subjected to corona treatment under optimised conditions (2 h after oviposition for 1 min). The results confirmed that this optimised treatment strategy prevented diapause in all the Chinese (DT and 932) and Japanese (BB and 7532) lineages (Figure 4C and Supplementary Table S4). The average hatching rates of the larvae in these commercial parental strains reached 97.49%–98.57% following VECT, which was comparable to the effect of corona or HCl treatment in preventing the diapause of eggs at 20 h after oviposition. In addition, we found by comparison that there was no obvious difference in the various physiological indexes of silkworms hatched by VECT and the traditional HCl treatment at 20 h after oviposition (Supplementary Table S5).

### Combination of the Very Early Corona Treatment Strategy and Pre-blastoderm Microinjection Mediated Germline Transformation of Diapause Silkworm Strains

**FIGURE 5:**
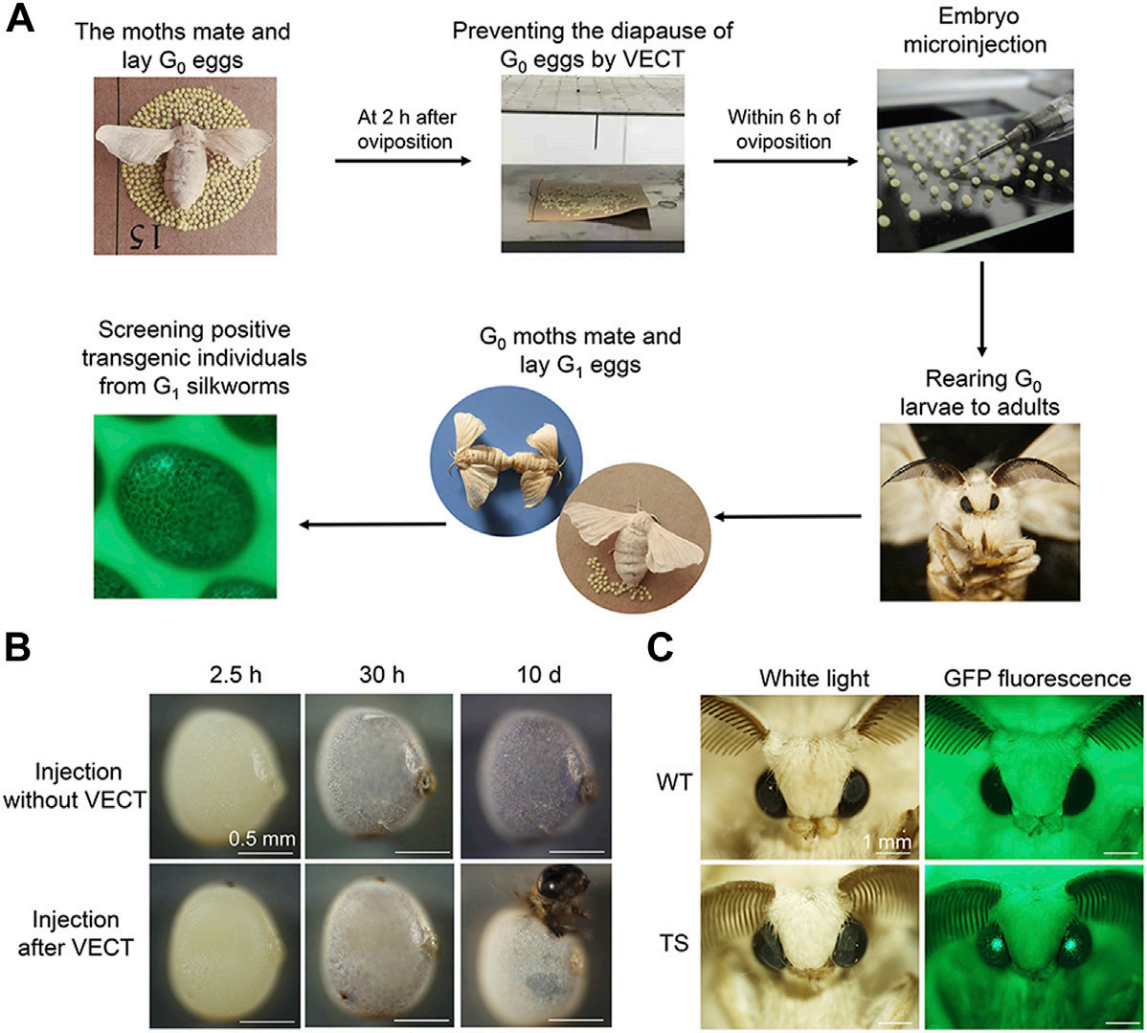
Germline transformation of diapause silkworm strains based on the VECT strategy combined with pre-blastoderm microinjection. **(A)** Schematic diagram of the experimental procedures for germline transformation of diapause silkworm strains. **(B)** Images of the developmental stages of G0 DZ eggs without or with VECT after injection of *piggyBac*-derived vectors. **(C)** Expression of the *EGFP* gene in adult G1 transgenic silkworms. Adults of the wild-type DZ (WT) and G1 transgenic silkworms (TS) showing a white light, and GFP fluorescence in the compound eye.

The eggs collected from each of the Chinese (DZ, DT and 932) and Japanese (BB and 7532) lineage strains at 2 h after oviposition were subjected to corona treatment for 1 min, as the optimum VECT strategy, to prevent diapause of eggs. The experimental procedures for germline transformation of diapause silkworm strains mediated by the combination strategy of VECT and pre-blastoderm microinjection are shown in Figure 5A. Eggs that were not subjected to the VECT were considered the control. As shown in Figure 5B, the colour of the injected G0 DZ eggs without VECT deepened gradually and eventually entered diapause. In contrast, diapause of the injected G0 DZ eggs was successfully prevented by VECT and the larvae hatched on day 10 of embryonic development. Finally, different numbers of GFP-positive G1 broods were obtained, and the rates of successful transgenesis for G1 broods varied from 3.37% to 16.18% for the different silkworm strains (Table 1). The expression of the *EGFP* gene in the embryos, larvae and adults from G1 broods of the DZ strain is shown in Supplementary Figure S1 and Figure 5B. The above results confirmed the feasibility of the combination strategy of VECT and pre-blastoderm microinjection in the transgenesis of silkworm diapause strains, especially bivoltine strains.

**TABLE 1:** Statistical results of injecting a mixture of pBac and pHA3PIG vectors into silkworm embryos of different strains.

Strain	Selected strategy for germline transformation	Injected eggs	Hatched eggs (%)^a^	G1 broods	G1 broods with GFP-positive larvae (%)^b^
DZ	Control^c^	423	0 (0)	0	0 (0)
DZ	VECT^d^	386	174 (45.08)	76	4 (5.26)
DT	VECT	374	135 (36.1)	57	5 (8.77)
BB	VECT	448	215 (47.99)	68	11 (16.18)
932	VECT	432	246 (56.94)	89	3 (3.37)
7532	VECT	398	150 (37.69)	54	7 (12.96)

a Percentage of (Number of hatched eggs)/(Number of injected eggs).

b Percentage of (Number of G1 broods with GFP-positive larvae)/(Number of G1 broods).

c Eggs that were not subjected to any treatment were considered the control.

d Optimised VECT, strategy, eggs at 2 h after oviposition were subjected to corona treatment (voltage, 12 kV; pole pitch, 8 mm) for 1 min, followed by microinjection.

## Discussion

Corona is a technology that uses a high-voltage direct-current power supply, comb needle cathode and a flat anode to produce a conical discharge at the needle tip. Researchers from Japan have discovered that electric induction treatment can result in the artificial incubation of silkworm eggs; therefore, corona stimulation to terminate the diapause of silkworms gradually became a hot spot in Chinese sericulture research in the 1990s. Compared with the widely used HCl or/and cold storage treatment in sericulture, the corona method has the advantages of procedural simplicity, low labour intensity and easy mechanisation. More importantly, terminating the diapause of silkworm eggs by corona treatment is a green, safe, and environmentally friendly artificial incubation method. The treatment completely avoids the need for the corrosive concentrated HCl, thereby effectively eliminating environmental pollution and avoiding physical damage to the operators.

The novel artificial corona instrument used in this study includes a creatively designed single-needle electrode as the discharging cathode rather than the comb-shaped ones used in previous studies (Ye et al., 1996). The corona apparatus with multi-needle electrodes is associated with problems such as unstable and uneven discharge of the plate during practical applications. In the preliminary experiment, we compared the effect of corona treatment using multi-needle electrodes or single-needle electrode in preventing the diapause of newly laid silkworm eggs, and also observed the phenomenon of non-uniform hatching of silkworm eggs due to uneven discharge of multi-needle electrodes, while a single-needle electrode can overcome the above shortcomings (Supplementary Figure S2). Under certain voltage conditions (12 kV used in this study), the single-needle electrode design makes the corona discharge more concentrated (a circular area approximately 44 mm in diameter). Therefore, the efficacy of terminating the diapause of silkworm eggs was better. Here it should be pointed out that the circular area with a larger corona diameter range can be theoretically obtained by adjusting the parameters of the corona instrument, such as the pole distance, the length of the metal pole needle, and the distance between two electrode metal plates. In this study, the hatching rates of the silkworm larvae within the effective range of the corona were almost 100%. Previous studies have confirmed that corona treatment can terminate diapause in newly laid and refrigerated eggs (Yang et al., 1994; Chen et al., 1995b). However, due to the limitation of early technical conditions, the power of the corona instruments used was low and was affected by factors such as the environmental condition, silkworm strains and age of the eggs. Moreover, the hatching of the larvae after corona treatment reported in previous studies was extremely unstable (Xu and Yang, 1998). Using the new corona instrument with a certain polar distance and input voltage, only 2 minutes were required to obtain hatching rates of more than 96% for newly laid eggs at 20 h after oviposition and refrigerated diapausing eggs. Various Chinese and Japanese lineage diapause silkworm strains can be stimulated with the new instrument to achieve a comparable effect as the traditional HCl treatment (Supplementary Tables S1, S2).

Early studies confirmed that newly laid eggs subjected to corona treatment within 5–30 h of oviposition resulted in approximately 90% larval hatchability (Xu and Yang, 1998). The effect of corona treatment in preventing the diapause of silkworm eggs within 5 h after oviposition has not been reported so far. In this study, we systematically investigated the effect of corona treatment in preventing the diapause of silkworm eggs at a very early developmental stage (within 4 h after oviposition) and successfully created a VECT strategy to prevent the eggs from entering diapause. By optimising the conditions and using the VECT strategy, the larval hatching rate was over 95%, irrespective of the Chinese or Japanese lineage silkworm strains. After rearing and hatching the silkworms, it was confirmed that the VECT did not have any adverse effect on the growth and development of the hatching larvae. Combining the VECT strategy with previous studies, to effectively extended the operating time range to prevent the diapause of eggs by corona treatment (i.e. 2–30 h), thus improved the production efficiency of corona treatment in preventing the diapause of silkworm eggs. In addition, during the embryonic development of silkworms, karyokinesis occurs first, then followed by cytokinesis (Xiang et al., 2005). The germ cells of the adult silkworm are derived from primordial germ cells during early embryo development stages (Xiang et al., 2005). Therefore, microinjection of *piggyBac*-derived vectors must be performed before the formation of primordial germ cells, thus the inheritable transgenic individuals have been produced only when the transposon integration events had occurred in the primordial germ cells. In the conventional silkworm germline transformation protocol, transformation vectors are usually injected into pre-blastoderm embryos within 6 h of oviposition (Tamura et al., 2000; Zhao et al., 2012). The current transgenic technology of diapause silkworm strains mainly includes two strategies: 1) HCl treatment within 3 h of oviposition and 2) incubating mother eggs at a low temperature (15°C) to prevent diapause of daughter eggs followed by microinjecting the non-diapausing daughter eggs (Xu et al., 2011; Zhao et al., 2012). In the first strategy, the residual HCl solution may enter the silkworm eggs through the injection needle, resulting in a significantly reduced hatchability of the larvae (Xu et al., 2011). However, the low-temperature strategy takes a long time to create transgenic silkworms and is unsuitable to terminate diapause in Japanese lineage silkworm strains (Zhao et al., 2012). Therefore, the abovementioned strategies are not widely used. In addition, this study creatively combined the advantage of the VECT strategy, i.e. releasing diapause of silkworm eggs within 4 h after oviposition, with the pre-blastoderm microinjection-based germline transformation technology and successfully screened positive transgenic individuals from several diapause Chinese and Japanese lineage silkworm strains. Finally, we established the transgenic technology of diapause silkworm strains that has the characteristics of safety and short cycle and can effectively overcome the disadvantages of the conventional HCl or low-temperature treatments. Theoretically, the VECT strategy can be used to terminate the diapause of eggs in any silkworm strain, thus promoting the development of molecular breeding of practical/diapause silkworm strains and providing a reference for the germline transformation of other diapausing insect species.

Currently, the mechanism of corona treatment to disrupt the diapause of silkworm eggs is unclear. Some studies have speculated that the electric current may cause a conformational change in the diapause hormone in the embryo, leading to its inactivation, thereby inducing the embryo to disrupt diapause and initiate development (Yang et al., 1994). Other studies have speculated that the free radicals generated by corona discharge in silkworm eggs are one of the important factors that disrupt diapause (Ye et al., 1996). It has been reported that the mortality of silkworm eggs after corona treatment is low, which may be due to the disinfection and sterilisation effect of ozone in the air (Chen and Zhu, 1997). It is also one of the reasons why the efficacy of corona treatment in the hatching of the silkworm eggs is comparable with that of HCl treatment. In general, the novel artificial corona instrument and the VECT strategy developed in this study, not only contribute to improving the artificial corona hatching system of silkworm eggs and practical large-scale application but also provide the equipment and technical support for promoting the analysis of the molecular mechanism of diapause of silkworm eggs as well as Lepidoptera.

## Data Availability

The original contributions presented in the study are included in the article/Supplementary Material, further inquiries can be directed to the corresponding author.
