# Supplementary material for: Very Early Corona Treatment-Mediated Artificial Incubation of Silkworm Eggs and Germline Transformation of Diapause Silkworm Strains
Source: Front Bioeng Biotechnol. 2022 Feb 11;10:843543. doi: 10.3389/fbioe.2022.843543 (PMC8874202; doi:10.3389/fbioe.2022.843543)
Supplement: Supplementary file 1 [file DataSheet1.PDF]

Supplementary Material

Very early corona treatment-mediated artificial incubation of silkworm eggs and germline transformation of diapause silkworm strains

Yu-Li Zhang[1,‡], Yang Huang[2,3,‡], Ping-Yang Wang[1], Qiang Li[4], Li-Hui Bi[1], Ai-Chun Zhao[2], Zhong-Huai Xiang[2], Ding-Pei Long[2,*]

[1]Sericultural Research Institute, Guangxi Zhuang Autonomous Region, Nanning, Guangxi, P. R. China, [2]State Key Laboratory of Silkworm Genome Biology, Key Laboratory for Sericulture Functional Genomics and Biotechnology of Agricultural Ministry, Southwest University, Chongqing, P. R. China, [3]Department of Biology, Georgia State University, Atlanta, GA, United States, [4]Department of Mathematics and Statistics, Georgia State University, Atlanta, GA, United States

[*]**Correspondence:**

Ding-Pei Long, State Key Laboratory of Silkworm Genome Biology, Key Laboratory for Sericulture Functional Genomics and Biotechnology of Agricultural Ministry, Southwest University, Beibei District, Chongqing 400716, P. R. China. Email: dlong26@gsu.edu

[‡]These authors contributed equally to this work.

Supplementary Table 1. Statistics of the hatching rates of the newly laid eggs by corona or HCl treatment at 20 h after oviposition

Strain	Group	Treatment condition	Experiment No. (n = 3)						Average hatching rate ($\bar{x}$ ± S.D., %)
			1		2		3		
			Treated eggs	Hatched eggs (%)†	Treated eggs	Hatched eggs (%)	Treated eggs	Hatched eggs (%)	
DZ	1#	Control‡	132	0 (0)	117	2 (1.71)	120	2 (1.67)	1.13 ± 0.8
	2#	Corona treated§	114	111 (97.37)	104	100 (96.15)	127	121 (95.28)	96.27 ± 0.86
	3#	HCl treated¶	123	118 (95.93)	111	108 (97.3)	138	131 (94.93)	96.05 ± 0.97
DT	1#	Control	165	0 (0)	173	2 (1.16)	147	1 (0.68)	0.61 ± 0.48
	2#	Corona treated	153	145 (94.77)	162	159 (98.15)	132	128 (96.97)	96.63 ± 1.4
	3#	HCl treated	148	140 (94.59)	184	182 (98.91)	154	148 (96.1)	96.53 ± 1.79
BB	1#	Control	156	0 (0)	148	0 (0)	172	0 (0)	0
	2#	Corona treated	142	137 (96.48)	137	132 (96.35)	159	157 (98.74)	97.19 ± 1.1
	3#	HCl treated	163	159 (97.55)	151	148 (98.01)	145	145 (100)	98.52 ± 1.06
932	1#	Control	125	0 (0)	132	3 (2.27)	139	1 (0.72)	1 ± 0.95
	2#	Corona treated	131	131 (100)	115	112 (97.39)	146	145 (99.32)	98.9 ± 1.11
	3#	HCl treated	121	120 (99.17)	124	122 (98.39)	148	146 (98.65)	98.74 ± 0.32
7532	1#	Control	174	0 (0)	155	0 (0)	160	0 (0)	0
	2#	Corona treated	167	164 (98.2)	148	145 (97.97)	142	138 (97.18)	97.78 ± 0.44
	3#	HCl treated	161	159 (98.76)	137	135 (98.54)	138	136 (98.55)	98.62 ± 0.1

†Percentage of (Number of hatched eggs)/(Number of treated eggs); ‡Without any treatment; §Treated with corona (voltage, 12 kV; pole pitch, 8 mm) for 2 min at RT; ¶Treated with HCl solution (specific gravity, 1.075) for 5 min at 46°C.

Supplementary Table 2. Statistics of the hatching rates of the refrigerated eggs after stored at 4°C for 60 days by corona or HCl treatment

Strain	Group	Treatment condition	Experiment No. (n = 3)						Average hatching rate ($\bar{x}$ ± S.D., %)
			1		2		3		
			Treated eggs	Hatched eggs (%)†	Treated eggs	Hatched eggs (%)	Treated eggs	Hatched eggs (%)	
DZ	1#	Control‡	154	0 (0)	138	0 (0)	112	0 (0)	0
	2#	Corona treated§	148	145 (97.97)	128	128 (100)	138	134 (97.1)	98.36 ± 1.22
	3#	HCl treated¶	159	154 (96.86)	147	143 (97.28)	129	128 (99.22)	97.79 ± 1.03
DT	1#	Control	162	0 (0)	177	0 (0)	173	0 (0)	0
	2#	Corona treated	174	168 (96.55)	156	153 (98.08)	177	172 (97.18)	97.27 ± 0.63
	3#	HCl treated	183	178 (97.27)	161	161 (100)	165	162 (98.18)	98.48 ± 1.13
BB	1#	Control	162	0 (0)	137	0 (0)	155	0 (0)	0
	2#	Corona treated	173	170 (98.27)	142	139 (97.89)	153	149 (97.39)	97.85 ± 0.36
	3#	HCl treated	180	180 (100)	146	143 (97.95)	147	142 (96.6)	98.18 ± 1.4
932	1#	Control	150	0 (0)	163	0 (0)	132	0 (0)	0
	2#	Corona treated	152	148 (97.37)	169	162 (95.86)	138	134 (97.1)	96.78 ± 0.66
	3#	HCl treated	167	163 (97.6)	142	133 (93.66)	121	117 (96.69)	95.98 ± 1.68
7532	1#	Control	179	0 (0)	133	0 (0)	155	0 (0)	0
	2#	Corona treated	159	153 (96.23)	148	144 (97.3)	159	156 (98.11)	97.21 ± 0.77
	3#	HCl treated	153	146 (95.42)	134	132 (98.51)	172	165 (95.93)	96.62 ± 1.35

†Percentage of (Number of hatched eggs)/(Number of treated eggs); ‡Eggs that were not subjected to any treatment were considered the control; §Eggs were subjected to corona treatment (voltage, 12 kV; pole pitch, 8 mm) for 2 min at RT; ¶Eggs were subjected to HCl treatment (specific gravity, 1.092) for 5 min 30 sec at 47.8°C.

Supplementary Table 3. Statistics of the average hatching rates of the newly laid DZ eggs within 4 h of oviposition by VECT

Length of corona treatment time	Average hatching rate of different developmental stage of eggs when corona treated ($\bar{x}$ ± S.D., %)							
	0.5 h	1 h	1.5 h	2 h	2.5 h	3 h	3.5 h	4 h
5 s	45.53 ± 5.55e	62.61 ± 3.55d	68.21 ± 4.03d	83.83 ± 3.88c	87.36 ± 4.24c	85.44 ± 5.11c	86.73 ± 2.48c	88.38 ± 6.54c
10 s	56.78 ± 4.71d	63.72 ± 5.39d	70.52 ± 4.5c	87.14 ± 4.54b	92.51 ± 1.53b	90.08 ± 3.04b	93.23 ± 3.24a	92.37 ± 4.22b
30 s	79.42 ± 6.88b	54.69 ± 8.59e	83.27 ± 5.38b	94.72 ± 2.68a	96.32 ± 2.04a	97.15 ± 1.76a	93.24 ± 1.78a	94.31 ± 2a
1 min	90.91 ± 2.83a	85.59 ± 6.88b	92.55 ± 3.4a	95.83 ± 3.13a	97.04 ± 1.35a	96.72 ± 1.12a	94.26 ± 2.38a	97.13 ± 0.79a
1 min 30 s	91.23 ± 2.27a	88.57 ± 1.64b	93.19 ± 2.76a	96.72 ± 2.11a	95.62 ± 2.3a	94.5 ± 1.56a	92.17 ± 3.18a	96.6 ± 1.72a
2 min	66.64 ± 3.72c	91.54 ± 1.73a	85.2 ± 3.21b	94.5 ± 1.98a	96.28 ± 1.7a	95.77 ± 0.74a	94.25 ± 4.28a	95.79 ± 2.01a
5 min	43.21 ± 2.94e	73.26 ± 3.21c	80.33 ± 5.23b	95.71 ± 3.7a	95.36 ± 2.27a	96.34 ± 1.23a	95.77 ± 1.33a	94.39 ± 2.38a
10 min	25.73 ± 7.12f	65.32 ± 4.24d	70.83 ± 3.43c	90.62 ± 2.03b	91.73 ± 1.57b	92.13 ± 2.17b	91.74 ± 3.94b	93.32 ± 3.25a

Note: Corona treatment was performed on all eggs based on the same parameters: voltage = 12 kV, distance between negative and positive plane electrodes = 18 mm, and pole pitch = 8 mm. Each group from one brood contained approximately 80–150 eggs. Different letters (a, b, c, d, e, and f) represent statistically significant difference (n = 3, *$P < 0.05$) among different length of corona treatment times on each developmental stage of eggs.

Supplementary Table 4. Statistics of the hatching rates of the newly laid eggs by corona treatment at 2 h after oviposition

Strain	Treatment condition	Experiment No. (n = 3)						Average hatching rate ($\bar{x}$ ± S.D., %)
		1		2		3		
		Treated eggs	Hatched eggs (%)†	Treated eggs	Hatched eggs (%)	Treated eggs	Hatched eggs (%)	
DT	Control‡	92	0 (0)	113	3 (2.65)	105	2 (1.9)	1.52 ± 1.12
	Corona treated§	97	95 (97.94)	105	101 (96.19)	117	116 (99.15)	97.76 ± 1.22
BB	Control	86	0 (0)	94	0 (0)	128	0 (0)	0
	Corona treated	96	96 (100)	117	113 (96.58)	142	140 (98.59)	98.39 ± 1.4
932	Control	126	1 (0.79)	103	0 (0)	111	2 (1.8)	0.86 ± 0.73
	Corona treated	116	112 (96.55)	125	125 (100)	98	94 (95.92)	97.49 ± 1.79
7532	Control	98	0 (0)	132	0 (0)	104	0 (0)	0
	Corona treated	105	103 (98.1)	125	122 (97.6)	93	93 (100)	98.57 ± 1.03

†Percentage of (Number of hatched eggs)/(Number of treated eggs); ‡Eggs that were not subjected to any treatment were considered the control; §Eggs were subjected to corona treatment (voltage, 12 kV; pole pitch, 8 mm) for 1 min at RT. Each group from one brood contained approximately 80–150 eggs.

Supplementary Table 5. The vitality traits of the larvae hatched by VECT or HCl treatment from different strains

Strain	Treatment condition	Duration of the 5th instar	Duration of all instars	Incidence rate of larvae ($\bar{x}$ ± S.D., %)	Cocooning rate ($\bar{x}$ ± S.D., %)	Rate of dead cocoons ($\bar{x}$ ± S.D., %)	Larva-pupa rate ($\bar{x}$ ± S.D., %)
DT	HCl treated†	7 d 3 h	21 d	2.45 ± 0.54	96.72 ± 3.28	3.17 ± 0.78	95.72 ± 0.24
	VECT‡	7 d 3 h	21 d	2.66 ± 0.47	97.82 ± 2.5	2.95 ± 0.71	96.23 ± 0.56
BB	HCl treated	7 d 12 h	21 d 6 h	3.02 ± 0.87	94.12 ± 3.13	6.21 ± 0.45	93.81 ± 1.62
	VECT	7 d 12 h	22 d 8 h	3.27 ± 0.91	95.77 ± 4.08	6.91 ± 0.82	94.78 ± 2.37
932	HCl treated	7 d 6 h	22 d 5 h	1.03 ± 0.34	98.03 ± 1.75	1.42 ± 0.68	96.23 ± 2.56
	VECT	7 d 6 h	22 d 5 h	1.17 ± 0.42	98.84 ± 0.74	1.95 ± 0.53	97.88 ± 1.27
7532	HCl treated	7 d 3 h	23 d 2 h	2.71 ± 0.81	96.28 ± 2.54	3.30 ± 0.82	95.79 ± 2.05
	VECT	7 d 2 h	23 d 1 h	1.95 ± 0.25	94.89 ± 3.67	2.14 ± 0.37	93.84 ± 3.28

†Eggs at 20 h after oviposition were subjected to HCl treatment (specific gravity, 1.075) for 5 min at 46°C; ‡Optimised VECT strategy, eggs at 2 h after oviposition were subjected to corona treatment (voltage, 12 kV; pole pitch, 8 mm) for 1 min at RT.

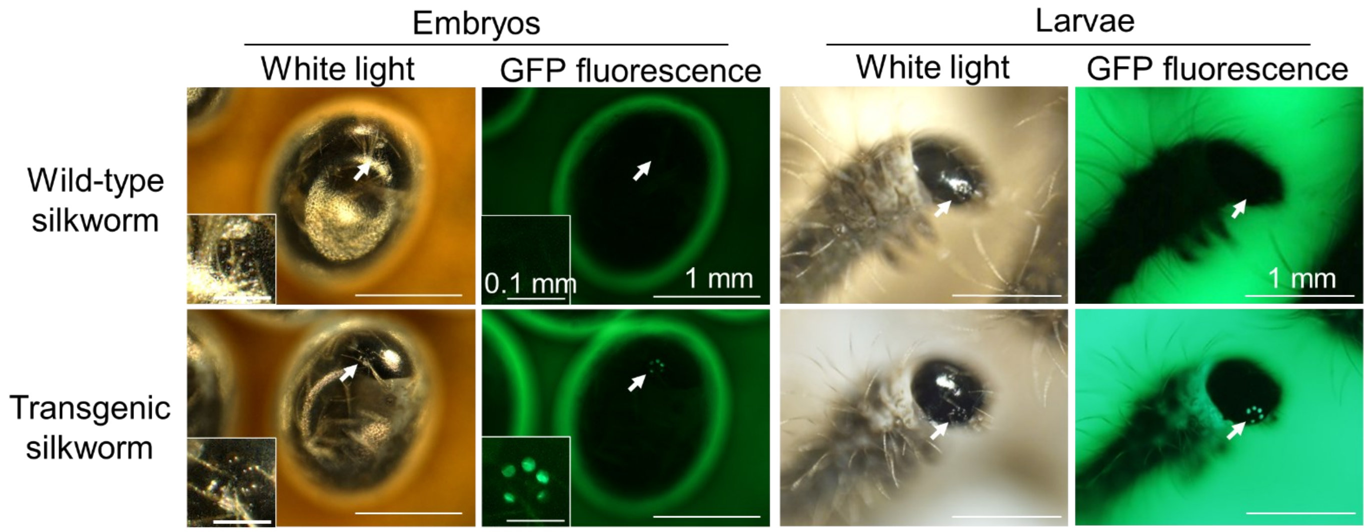

Supplementary Figure 1. Expression of the *EGFP* gene in the embryos and larvae of the G1 transgenic silkworms. Ten-day-old embryos and newly hatched larvae of wild-type silkworms and G1 transgenic silkworms showing a white light, and GFP fluorescence in developing larval ocelli (white arrowheads).

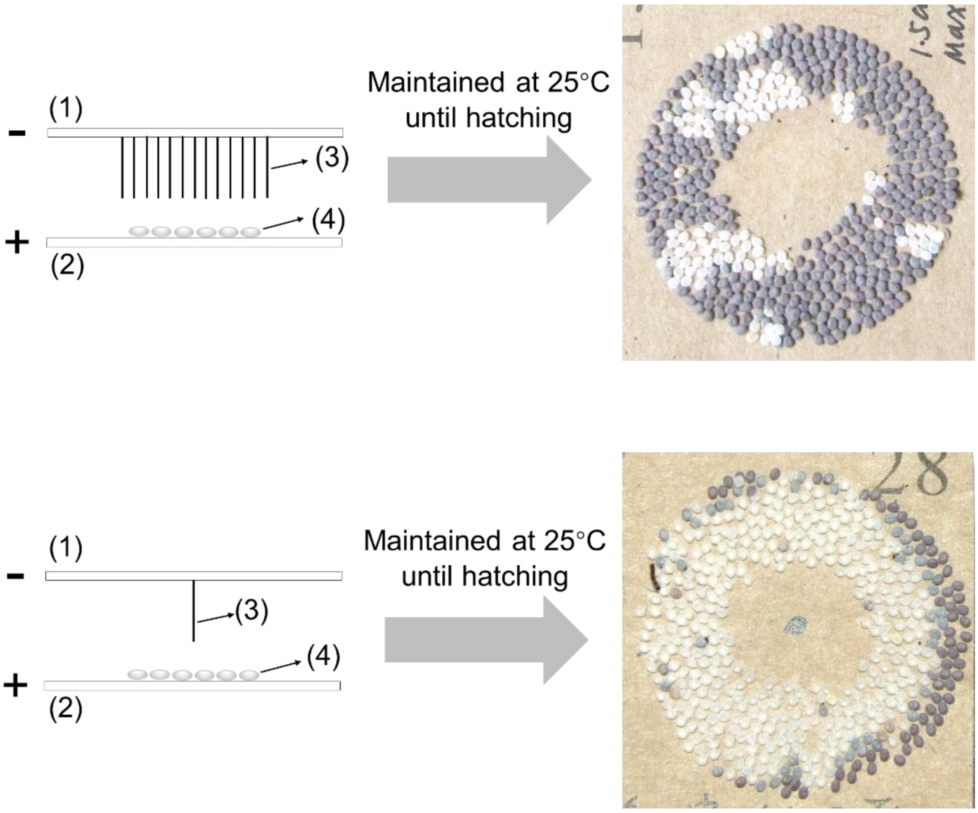

Supplementary Figure 2. Photographs of the effect of corona treatment using multi-needle electrodes (top) or single-needle electrode (bottom) in preventing the diapause of newly laid DZ eggs. (1) negative and (2) positive electrode metal plates; (3) metal pole needle; (4) Kraft paper covered with eggs.
